# Ozanimod to Treat Relapsing Forms of Multiple Sclerosis: A Comprehensive Review of Disease, Drug Efficacy and Side Effects

**DOI:** 10.3390/neurolint12030016

**Published:** 2020-12-03

**Authors:** Grace Lassiter, Carlie Melancon, Tyler Rooney, Anne-Marie Murat, Jessica S. Kaye, Adam M. Kaye, Rachel J. Kaye, Elyse M. Cornett, Alan D. Kaye, Rutvij J. Shah, Omar Viswanath, Ivan Urits

**Affiliations:** 1 School of Medicine, Georgetown University, Washington, DC 20007, USA; 2 School of Medicine, Louisiana State University Shreveport, Shreveport, LA 71103, USA; cmela8@lsuhsc.edu (C.M.); troone@lsuhsc.edu (T.R.); amurat@lsuhsc.edu (A.-M.M.); 3 Department of Pharmacy Practice, Thomas J. Long School of Pharmacy and Health Sciences, University of the Pacific, Stockton, CA 95211, USA; j_kaye1@u.pacific.edu (J.S.K.); akaye@pacific.edu (A.M.K.); 4 School of Medicine, Medical University of South Carolina, Charleston, SC 29425, USA; rachelkaye17@hotmail.com; 5 Department of Anesthesiology, Louisiana State University Shreveport, Shreveport, LA 71103, USA; ecorne@lsuhsc.edu (E.M.C.); akaye@lsuhsc.edu (A.D.K.); rshah2@lsuhsc.edu (R.J.S.); viswanoy@gmail.com (O.V.); ivanurits@gmail.com (I.U.); 6 Department of Neurology, Louisiana State University Shreveport, Shreveport, LA 71103, USA; 7 College of Medicine-Phoenix, University of Arizona, Phoenix, AZ 85724, USA; 8 Department of Anesthesiology, School of Medicine, Creighton University, Omaha, NE 68124, USA; 9 Valley Anesthesiology and Pain Consultants—Envision Physician Services, Phoenix, AZ 85004, USA; 10 Southcoast Health, Southcoast Physicians Group Pain Medicine, Wareham, MA 02571, USA

**Keywords:** ozanimod, multiple sclerosis, sphingosine-1-phosphate receptor modulator, chronic pain

## Abstract

Multiple sclerosis (MS) is a prevalent and debilitating neurologic condition characterized by widespread neurodegeneration and the formation of focal demyelinating plaques in the central nervous system. Current therapeutic options are complex and attempt to manage acute relapse, modify disease, and manage symptoms. Such therapies often prove insufficient alone and highlight the need for more targeted MS treatments with reduced systemic side effect profiles. Ozanimod is a novel S1P (sphingosine-1-phosphate) receptor modulator used for the treatment of clinically isolated syndrome, relapsing–remitting, and secondary progressive forms of multiple sclerosis. It selectively modulates S1P1 and S1P5 receptors to prevent autoreactive lymphocytes from entering the CNS where they can promote nerve damage and inflammation. Ozanimod was approved by the US Food and Drug Administration (US FDA) for the management of multiple sclerosis in March 2020 and has been proved to be both effective and well tolerated. Of note, ozanimod is associated with the following complications: increased risk of infections, liver injury, fetal risk, increased blood pressure, respiratory effects, macular edema, and posterior reversible encephalopathy syndrome, among others. Further investigation including head-to-head clinical trials is warranted to evaluate the efficacy of ozanimod compared with other S1P1 receptor modulators.

## 1. Introduction

Multiple sclerosis (MS) is one of the most prevalent and disabling neurologic conditions worldwide. It is a chronic inflammatory disease that results in widespread neurodegeneration and the formation of focal demyelinating plaques in the white and grey matter of the CNS [1,2,3]. MS globally affects approximately 2.5 million people, with the majority aged 20–40 years when symptoms present [4]. Women are affected more often than men, with a female to male prevalence ratio of almost 3:1 [5]. To date, there has not been one specific factor identified as the cause of disease; rather, MS is thought to arise in genetically susceptible individuals who are exposed to environmental and immune triggers [3]. Environmental and lifestyle factors such as Vitamin D deficiency, low sun exposure, Epstein–Barr virus, smoking, and obesity have shown to be involved in the development of MS [6].

The onset of MS symptoms is usually sudden and most commonly presents as unilateral loss of vision, sensory loss, motor and muscle weakness, or ataxia [4,7]. Diagnosis of MS is established using clinical judgment and neurologic examinations, with evidence of CNS damage disseminated in time and space. Magnetic resonance imaging (MRI) with gadolinium is useful in identifying demyelinated lesions throughout the CNS. Cerebrospinal fluid analysis and immunoglobulin levels are also helpful in establishing diagnosis [8].

Multiple sclerosis is classified into clinically isolated syndrome (CIS), relapsing–remitting MS (RRMS), secondary progressive MS (SPMS), primary progressive MS (PPMS) and progressive relapsing MS (PRMS) based on the clinical course of disease [6]. CIS is identified as the first episode of symptoms, with MRI findings similar to MS but not meeting full diagnostic criteria. RRMS is the most common form and constitutes eighty-five percent of all MS patients, characterized by recovery periods between attacks [6,9]. Most people with RRMS will convert to the secondary-progressive course (SPMS), with continued worsening of symptoms. Primary-progressive MS (PPMS) is defined by worsening symptoms from the disease onset without periods of relapse [6].

The pathophysiology of MS has traditionally been viewed as a two-stage process, with focal inflammation and demyelination early in the course, and axonal loss and neurodegeneration more prominent over time [8]. The disease pathology begins when autoreactive T-cells cross the blood-brain barrier (BBB) and induce inflammation. These CD8+ T-cells secrete pro-inflammatory cytokines and aid in the activation and recruitment of other immune cells, including B-cells, macrophages, microglia, astrocytes, and plasma cells [6].

There is no current cure for MS, but there are several disease-modifying therapies (DMTs) used, especially for the relapsing–remitting MS [10]. Although the currently approved pharmacologic options have demonstrated efficacy in managing and slowing the disease, they are associated with greater risks and non-compliance due to side effect profiles [4]. This emphasizes the continued need for novel medications with high safety profiles, particularly those that target MS early in its course to mitigate disease progression and improve quality of life.

## 2. Multiple Sclerosis Epidemiology

Several environmental, genetic, and immunologic risk factors have been studied in relation to MS susceptibility and disease prevalence [11]. Epstein–Barr virus (EBV) has been shown to increase susceptibility to developing MS, with molecular mimicry and EBV-induced B-cell immortalization as possible explanations for this relationship [10,12,13,14]. Smokers have a higher overall risk of MS and a higher association with transformation into secondary progressive MS once the disease is established [6].

The mean age of peak incidence of MS in adults is 30 years, with the onset of MS in females approximately 1–5 years before the onset in men [6,9]. In fact, females are almost three times as likely as males to be affected by MS. The female to male prevalence ratio of MS in the United States was estimated to be 2.8:1 [5]. Similarly, a study performed in Denmark reported that the incidence of MS in women has doubled over the last six decades, while the incidence in men increased by only 24% [15].

## 3. Risk Factors

Many ecologic studies have shown a positive correlation of MS with increased latitudes and a negative correlation with ultraviolet radiation exposure. Migration studies, reduced UVB exposure, and vitamin D deficiency have been explored as potential explanations for these correlations [16,17,18]. New Mendelian randomization analyses reveal evidence of a causal relationship between low vitamin D and susceptibility to developing MS [17,19,20].

A large 2019 case–control study analyzed sun exposure and MS risk in three countries where MS is prevalent. The study found a 50% increased risk of developing MS in the group with the lowest amount of accumulated sun exposure [21]. Additionally, when comparing groups categorized by sun-seeking and sun-avoiding behavior, the sun-avoiding group had a 75% higher risk of developing MS [21,22,23]. Several recent studies have described potential mechanisms of the protective role that UVB exposure has against MS. In addition to directly activating vitamin D, UVB light also plays an immunoregulatory role independent of vitamin D by activating regulatory T and B cells, leading to suppression of cell-mediated immunity [24,25].

Although MS is not currently considered a heritable disease, several recent studies suggest the existence of familial MS (FMS). A meta-analysis from 2018 found a pooled prevalence of FMS in the MS population of 12.6% by random effect, highlighting the interplay between genetics and the environment on disease development. Several studies suggest a positive family history in nearly 20% of patients with MS [6,10,26]. Furthermore, one of the strongest known genetic associations with multiple sclerosis is the HLA-DRB1*15:01 allele [10,27]. The association of HLA class II polymorphisms with MS may be related to gene expression, with vitamin D thought to regulate DRB1*1501 expression via a vitamin D response element (VDRE) in the promoter region of the gene [17,27]. Moreover, a systematic review of prior analyses investigating HLA-DRB1 and HLA-DQB1 polymorphisms determined that DRB1*15 and DQB1*06:02 alleles are associated with an increased risk of MS across numerous ethnicities, and identified other alleles protective of disease [28,29,30,31,32]. This highlights the predictive potential of genetic factors on MS prevalence in families.

Recently, Mendelian randomization studies have provided evidence that BMI and physical activity have causal relationships with MS and recommend exercise as a preventative and therapeutic intervention [33].

## 4. Pathophysiology

Multiple sclerosis is an autoimmune disorder with pathology characterized by perivenular inflammatory lesions, demyelination, astrocytic scarring, and neuroaxonal damage [3,10]. The inflammatory lesions are first formed when peripheral immune cells migrate into the CNS through the blood-brain barrier [4,6]. Early in the disease, such as in acute RRMS, CD8+ T-cells and B-cells are the major types of cells to infiltrate the BBB and initiate the formation of focal white matter lesions in the brain and spinal cord. Activated macrophages and microglia form plaques, characterizing myelin, and oligodendrocyte destruction [3,34]. Some neuroaxonal loss may be seen in this phase as well, though it is more prominent in progressive courses of MS [1,6].

As the disease progresses, diffuse and ongoing axonal injury ensues, even in areas with normal myelin, and self-perpetuating neurodegeneration leads to diffuse white and grey matter atrophy [3,35]. A milder inflammatory pattern within the plaques is seen in the progressive courses of MS, along with microglial activation near the periphery and a slower pace of expansion [6,36].

Recent studies suggest B-cells play a role in the pathogenesis of MS through an antigen-driven B-cell response. B-cells in the CNS of patients with MS secrete oligoclonal immunoglobulin bands (OCBs), which are found in 95% of MS patients in their first clinical presentation. OCBs are highly sensitive prognostic indicators for the future development of MS. They also predict a more aggressive disease course than seen in patients without OCBs [37,38,39]. While several autoreactive immunoglobins have been identified in MS, the pathogenicity of OCBs remains unsupported [39,40]. It has thus been suggested that B-cells contribute to the pathophysiology of MS through antigen presentation, which induces and activates CD4+ helper T-cell proliferation. IL-6 producing B-cells influence pro-inflammatory cytokine production, leading to differentiation to Th17, an autoimmune driving T-cell. In contrast, IL-10 producing B-cells secrete anti-inflammatory cytokines, which may play a role in the remitting phase of RRMS [39]. Therapies that target B-cell surface markers, such as CD19 and CD20, have shown success as potential treatments for MS by delaying relapse rates and slowing disease progression [39].

## 5. Presentation

The disease course of multiple sclerosis varies significantly between different patients, and a single patient can experience changes in their MS phenotype over time [10,41]. The onset of MS typically begins with a sudden loss of some neurologic function. Patients can present with a broad range of one or more symptoms with varying severity, reflective of the spatial distribution of lesions affecting different areas in the central nervous system [4,10,18]. Common symptoms include optic neuritis, double vision, bladder and bowel dysfunction, fatigue, ataxia, and sensory and motor dysfunction [4,10,42]. Clinically isolated syndrome (CIS) is defined as the initial disease presentation with signs of inflammatory demyelination suspect of MS, but not yet fulfilling diagnostic criteria of dissemination in time [41]. The majority of patients present around age 30–40 and follow the relapsing–remitting disease course, where they experience exacerbations of new or increasing symptoms followed by phases of varying recovery. Relapsing–remitting MS can transform into secondary progressive MS, typically around a decade after disease onset [1,41]. Patients can also follow the primary progressive MS course, where they typically present around age 50 and suffer from progressively worsening symptoms with no episodes of attacks or remission [1,41,43,44].

## 6. Current Treatment of Multiple Sclerosis

The treatment of multiple sclerosis has increased in complexity over the decades. The current therapeutic options include three broad categories: management of acute relapse, disease-modifying therapies (DMTs), and symptomatic management [45,46].

### 6.1. Management of Relapses

An MS relapse is characterized as inflammation along the nerve and/or myelin sheath creating new symptoms or exacerbating existing symptoms. The goals in managing an acute relapse include increasing time to functional recovery, decreasing the intensity of the attack, and reducing enduring neurologic deficits [47]. In moderate to severe acute relapses, daily corticosteroids (500–1000 mg of methylprednisolone) can be used to decrease the duration of attack [46]. In cases of severe relapses, plasma exchange is sometimes used [46]. Although rare, relapses during pregnancy can be safely treated with intravenous immunoglobulin [45]. ACTH is also an option for patients with inadequate venous access, those who favor self-injection, or patients resistant to corticosteroids [45].

### 6.2. Disease-Modifying Therapy (DMT)

DMTs aim to avoid long-term disability by targeting the myelin and axonal sheath destruction seen in MS [48]. DMTs have been shown to reduce disability in relapsing–remitting MS, but have shown little value in progressive disease courses [45,49]. DMTs are currently available via three routes of administration: self-injected, oral, and intravenous [45].

#### 6.2.1. DMTs: Self-Injected

The self-injected DMTs consists of Interferon Beta-1a, Interferon Beta-1b, Peginterferon Beta-1a, and Glatiramer Acetate (GA). There are four types of interferon formulations (Betaseron, Extavia, Rebif, Avonex) in addition to a pegylated form with a long-circulating half-life. Each drug works by type 1 interferon stimulation [50]. Interferon-beta decreases the intrusion of the blood-brain barrier and acts on T-cells, B-cells, and cytokines, whereas GA most likely regulates regulatory T-cells [51]. Despite the rise of oral therapies, IFN and GA remain the drugs of choice for many clinicians due to their relatively safe drug profiles [52].

#### 6.2.2. DMTs: Oral

The oral DMTs consist of Dimethyl Fumarate (DMF), Fingolimod, Siponimod, Teriflunomide, and Cladribine. DMF (Tecfidera), originally approved for psoriasis management, was approved in 2013 for the treatment of RRMS [53,54,55]. DMF works via NRF2 activation and downregulation of NFkB [50]. It is thought to exert antioxidant effects that reduce neuronal death and the destruction of the myelin sheath in MS [56]. It is thought that DMF is neuroprotective by reducing transcription of reactive oxygen species through the modulation of NRF-2 in astrocytes, oligodendrocytes, and neurons [53].

Fingolimod (Gilenya) was approved in 2010 as the first sphingosine-1-phosphate receptor (S1PR) modulator for the treatment of RRMS. Fingolimod targets p38 MAP kinase, reduces excitotoxicity, and penetrates the blood-brain barrier to directly modulate the activity of CNS cells [55,57]. Fingolimod also reduces the expression of sphingosine-1-phosphate (S1P) on B-cells, effectively reducing their exit from lymph nodes and the spleen [57,58]. Clinical trials showed that compared with placebo, the annualized relapse rate (ARR) was decreased by 60% with 1.25-mg and 54% with 0.5-mg of Fingolimod. Treatment for 24 months with Fingolimod resulted in decreased gadolinium-enhancing lesions on MRI as well as a reduction in new or enlarging T2 lesions. Of note, after 24 months participants had significantly less brain volume loss compared with placebo [59,60,61]. Although Fingolimod was a novel drug and demonstrated efficacy on MS disease course, its non-selective modification of SP1R subtypes and long half-life were responsible for numerous undesirable effects, including bradycardia, atrioventricular block, and macular edema [61,62,63,64]. As such, patients must undergo first-dose monitoring for at least six hours when starting the drug.

Teriflunomide was first approved in 2012 for the treatment of CIS, RRMS, and active secondary progressive disease in adults. It is the active metabolite of Leflunomide, a drug approved for the treatment of moderate to severe rheumatoid arthritis. Teriflunomide works by inhibiting dihydrofolate dehydrogenase, leading to the rise in B and T-cells without causing cytotoxicity [65]. Inhibition of this enzyme leads to the cessation of de novo pyrimidine synthesis in proliferating cells [66]. Teriflunomide contains a black box warning due to hepatotoxicity but is otherwise well tolerated in MS patients [52,67].

Siponimod is a selective S1P1 and S1P5 receptor modulator which works by internalizing S1P1 receptors to reduce efflux of lymphocytes from the lymph nodes and thymus. This S1P receptor specificity may limit toxicity, namely cardiac arrythmias. However, results from a phase I trial indicate that Siponimod may cause dose-dependent and transient bradycardia due to activation of G-protein-coupled inwardly rectifying potassium (GIRK) channels in atrial myocytes [68]. Other adverse events include conduction abnormalities, elevated liver function tests, and macular edema. Patients must also undergo cytochrome P_450_2C9 (CYP2C9) genetic testing prior to starting Siponimod to determine the correct dosing and titration schedule. Results from a phase II trial indicate that Siponimod may be beneficial in patients with relapsing–remitting MS [69]. While results from phase III trials indicate its efficacy in secondary progressive MS, there is limited data validating Siponimod’s efficacy in progressive forms of the disease [70,71].

Cladribine is deoxyadenosine analogue prodrug indicated for the treatment of adults with highly active relapsing MS. A phase 3 trial CLARITY and its extension found that a cumulative Cladribine dose of 3.5 mg/kg administered in two courses one year apart reduced MRI-assessed lesion frequency, disability progression, and clinical relapse [72]. The drug was also found to improve quality of life (HR-QOL) versus placebo over the 96-week study [73]. Furthermore, the extension study found no further clinical benefit gained from continuing versus discontinuing the drug after the first two annual courses of treatment [74]. A recent post hoc analyses of CLARITY reported that Cladribine was more beneficial in patients with high disease activity (HDA) compared to patients without HDA [75].

#### 6.2.3. DMTs: Intravenous (IV)

The intravenous DMTs consist of Alemtuzumab, Natalizumab, Mitoxantrone, Ocrelizumab, and Daclizumab. The third-line therapy for MS, Alemtuzumab (Lemtrada), is a monoclonal antibody targeting CD52 [53]. Alemtuzumab induces widespread destruction of CD52-containing cells such as T-cells, B-cells, natural killer cells, dendritic cells, monocytes, and granulocytes [53]. Yet the combination of a prolonged CD27+ memory B-cell lymphopenia, depletion in peripheral T-cell counts, and increased serum B-cell activating factor (BAFF) may permit the development of antibody-mediated autoimmunity, such as Graves disease or autoimmune thrombocytopenia [76,77]. Of note, Alemtuzumab significantly reduces the rate of attacks in patients with relapsing forms of MS.

Natalizumab (Tysabri) is a monoclonal antibody targeting VLA-4, an integrin dimer located on T-cell membranes. The blockade of VLA-4 results in T-cell inability to adhere to the endothelium and migrate into the brain [78]. Although rare, patients being treated with Natalizumab for more than two years, and who have undergone chemotherapy or immunosuppressive therapy, are at increased risk for the activation of the John Cunningham virus (JCV) [46]. Because JCV can lead to progressive multifocal leukoencephalopathy, a progressive and often fatal inflammation of the white matter of the brain, MRI and JCV status monitoring are required while on Natalizumab [79].

Mitoxantrone (Novatrone) is a topoisomerase inhibitor often used to treat prostate cancer and certain types of leukemia (50). It is used to reduce the number of relapses in MS patients through intermittent immunosuppresion. Side effects include leukopenia, hair loss, nausea, vomiting, and increased risk of infections. Ocrelizumab (Ocrevus) was approved in 2017 for the treatment of relapsing and primary progressive forms of MS. Ocrelizumab acts via CD20+ B-cell depletion and is the first drug to successfully treat primary progressive forms of multiple sclerosis [53].

Rituximab (Rituxan) is a monoclonal antibody that also acts via CD20+ B-cell depletion and is used off-label for the treatment of MS. Rituximab is primarily used to treat lymphoma, RA, ANCA-associated vasculitis, and Neuromyelitis Optica. Clinical trials for MS showed that Rituximab add-on therapy demonstrated tolerability and was effective at significantly reducing gadolinium-enhancing brain lesions [80]. Daclizumab (Zinbryta) is a monoclonal antibody that binds CD25, the alpha unit of the IL-2 receptor [50]. Because IL-2 induces the growth of activated T-cells, Daclizumab acts as an immunosuppressant by inhibiting T-cell proliferation [81].

### 6.3. Neuromodulation

Neuromodulation is utilized in MS treatment through the use of intrathecal baclofen pumps, functional electrical stimulation, deep brain stimulation, transcranial magnetic stimulation, spinal cord stimulation, and bladder stimulation [82].

An intrathecal baclofen (ITB) pump is a device that is implanted in a patient’s subcutaneous tissue and terminates with a catheter in the intrathecal space of the spinal cord [83]. This device can reduce spasticity in MS by administering baclofen directly to the spinal cord. Continuous pump infusions of baclofen have shown to be more effective than oral baclofen [84]. Functional electrical stimulation can also prevent the weakening or atrophy of muscles, such as the foot extensors [82].

Deep brain stimulation (DBS) involves inserting electrodes deep within the brain and is commonplace in refractory cases of essential tremor and Parkinson’s disease [85]. Although DBS does not modify disease course in MS, it has been shown to reduce disabling symptoms such as tremor frequency, trigeminal neuralgia, chronic pain, and bladder dysfunction [82]. Similarly, transcranial magnetic stimulation (TMS) is a non-invasive technique that changes the magnetic field in order to induce an electric current to a focal area of the brain. TMS is mainly used in the treatment of stroke complications, neuropathic pain, and major depression. Of note, studies indicate that repetitive TMS may be useful for symptom mitigation, particularly the muscle and bladder spasticity commonly seen in MS patients [86,87,88].

Spinal cord stimulation (SCS) involves implanting a subcutaneous device that sends electrical currents to the epidural space of the spinal cord [89]. SCS may play a role in reducing the neuropathy, spasticity, and bladder dysfunction seen in MS, but studies showing the effects on motor function are inconclusive [90]. Furthermore, bladder stimulation is the technique of treating overactive bladder via the stimulation of nerves in the lower back or pelvic muscles. Sacral or posterior tibial nerve stimulation may provide an increased quality of life for MS patients suffering from bladder dysfunction [91,92,93].

## 7. Ozanimod Drug Information

In March 2020, the FDA approved Ozanimod for the treatment of RRMS, SPMS and CIS. Ozanimod, sold under the brand name ZEPOSIA, is a sphingosine-1-phosphate receptor (S1PR) modulator. Unlike earlier drugs of its class, Ozanimod is currently the only US FDA approved S1PR modulator that does not require genetic testing or first-dose observation [94,95].

Patients should undergo a series of baseline assessments, including complete blood count, electrocardiogram, and liver function tests, before starting Ozanimod. If the patient has a history of uveitis or macular edema, they should also receive an ophthalmic examination. As the drug may increase the risk of infections due to lymphocyte depletion, varicella-zoster virus antibodies should be tested for and patients should avoid live-attenuated vaccines during treatment [94,96].

Initiation of Ozanimod within six months of a cardiovascular event is contraindicated. Patients with a history of heart block, including Mobitz type II second or third-degree atrioventricular blocks, sino-atrial block, and sick sinus syndrome, should not be treated with Ozanimod unless they have a pacemaker. Other contraindications include severe untreated sleep apnea and simultaneous treatment with a monoamine oxidase inhibitor [94,96].

Ozanimod carries a risk of infections, bradyarrhythmia and atrioventricular conduction delays, liver injury, a decline in pulmonary function, transient decrease in heart rate, increased blood pressure, fetal risk, and macular edema. Adverse reactions from pooled SUNBEAM and phase III RADIANCE data include upper respiratory infection, elevated hepatic enzymes, orthostatic hypotension, urinary tract infection, hypertension, and back pain [94,96,97,98]. Despite these possible reactions, patients with relapsing multiple sclerosis reported good tolerability during phase II and II clinical trials [94,97,98,99]. Ozanimod has a relatively short half-life and lower peak plasma concentrations when compared to Fingolimod, the first S1PR modulator approved for the treatment of RRMS. These differences allow for once-daily dosing and contribute to lower systemic side effects [61,100].

## 8. Ozanimod Mechanism of Action

Ozanimod (Zeposia) is an immunomodulatory drug used for the treatment of clinically isolated syndrome, relapsing–remitting, and secondary progressive forms of MS. Ozanimod is an oral agent that modifies the course of the disease by selectively modulating sphingosine-1-phosphate receptor-1 (S1P1) and receptor-5 (S1P5) activity [59]. S1P is phosphorylated by sphingosine kinase 1 or 2 to become an active phospholipid [59]. Once active, these phospholipids, heavily concentrated in red blood cells, the brain, spleen, and eyes, regulate numerous functions involved in immunity, heart rate, smooth muscle tone, and endothelial cell development [57].

There are four types of sphingosine phosphate receptors. Type 1, 2, and 3 receptors exist ubiquitously. Type 4 and 5 receptors are present in lymphoid tissue, and the spleen and oligodendrocytes, respectively [59]. The expression of these receptors is low in lymph nodes, and expression allows for the exit of T-cells in response to the lymph–lymph node chemotactic gradient [59]. A strong S1P gradient is created by high concentrations in the blood and lymph and low concentrations in the intracellular and interstitial fluids [101]. B-cells and T-cells can use this gradient as a signal to enter the circulation. Disruption of this gradient results in lymphopenia due to lymphocyte failure to exit from lymphoid organs [102]. In fact, mice deprived of S1P1 show an absence of circulating lymphocytes as these cells remain confined to the thymus and other secondary lymphoid tissues [103].

S1P also holds an important function in the regulation of vascular integrity as a suppressor of angiogenesis [98]. These phospholipids reinforce the adherens junctions and stimulate the development of endothelial cells [104]. S1P localization and signaling are crucial for preserving vascular homeostasis, as it has been shown that mice that lack S1P and are fed a diet high in lipids have increased formation of atherosclerotic lesions [105].

S1P is hydrophobic and requires a chaperone molecule without which it cannot freely circulate in the bloodstream. Approximately 65% of S1P circulates bound to HDL-anchored ApoM, while the remaining S1P circulates bound to albumin [106]. S1P’s functions vary depending on whether it is bound to ApoM or albumin. Only S1P bound to ApoM was shown to suppress inflammatory responses induced by cytokines [105]. Of note, S1P concentration is reduced in coronary artery disease, acute myocardial infarction, type II diabetes, and chronic kidney disease [107,108,109,110,111,112]. Via the modulation of S1P1 and S1P5, Ozanimod prevents circulating autoreactive lymphocytes from entering the CNS from peripheral tissues, in addition to reducing their concentration in the bloodstream.

## 9. Pharmacokinetics

### 9.1. Absorption and Distribution

Ozanimod is an oral medication that is absorbed via the gastrointestinal tract with a peak concentration (C_max_) of 0.244 ng/mL occurring at 6 to 8 h after administration [111]. The volume of distribution of Ozanimod ranges from 73 to 101 L/kg [111]. After Ozanimod was given under a fasting state, the median T_max_ was 8.0 to 12.0 h for single does and 8.0 h for multiple doses [112]. Trials recognize a near-dose proportional increase in C_max_ and the area under the plasma–concentration–time curve (AUC) [112]. Food intake does not affect the exposure of Ozanimod and its active metabolites [113]. The drug can be taken without food.

### 9.2. Metabolism

Ozanimod undergoes metabolism via the cytochrome P_450_2C8 (CYP2C8) system of the liver, and to a lesser extent CYP3A4, to form its active metabolite. Renal clearance is not a vital excretion path for Ozanimod [112].

### 9.3. Elimination

Ozanimod, eliminated through the urinary tract, has a mean single oral dose excretion of 0.03% and 0.06%, a 7-day regimen mean excretion of 0.04% to 0.09%, and a 28-day regimen mean excretion of 0.03% to 0.06% [112]. Ozanimod was shown to have a single dose renal clearance range from 0.116 to 0.287 L/h, 7-day dosing regimen renal clearance range from 0.189 to 0.435 L/h, and a 28-day regimen renal clearance range from 0.229 to 0.291 L/h [112].

## 10. Pharmacodynamics

Ozanimod causes a prompt, dose-dependent, and reversible reduction in the absolute lymphocyte count at all dosing regimens studied (Table 1) [112]. This reduction in absolute lymphocytes has also been seen with other drugs in the class of S1P modulator [112]. These pharmacodynamic effects display a preference for lymphocyte subtypes with increased effects on CD4+ CCR7+ and CD8+ CCR7+ T-cells [112]. Decreased effects were noted on effector and central memory cells [112]. This lymphocyte subset selectivity has also been seen in other drugs that modulate S1P, and may indicate a class ability to maintain protective immunity while targeting the immunopathologic pathway of MS [112].

## 11. Clinical Studies: Safety and Efficacy

### 11.1. Phase I Studies

A phase I randomized, double-blind, placebo-controlled trial investigated the safety of ozanimod in 88 healthy volunteers (Table 1). A single ascending dose (SAD) cohort was assigned doses of 0.3, 1, 2, or 3 mg once daily; a 7-day multiple ascending-dose (MAD-7) cohort was assigned doses of 0.3, 1, or 2 mg once daily; a 28-day multiple ascending-dose (MAD-28) cohort was assigned doses of 0.3, 1, or 1.5 mg once daily; a dose-escalation (DE) cohort was given 0.3 mg once daily on day 1–3, 0.6 mg once daily on days 4–5, 1 mg once daily on days 6–7, and 2 mg once daily on days 8–10. The most common treatment-emergent adverse effect (TEAE) was contact dermatitis from ECG pads. Participants receiving ozanimod showed greater reductions in mean hourly heart rate compared with placebo. This effect appeared to be dose-dependent, with the largest reduction in participants receiving ozanimod 3 mg. The DE cohort showed heart rate reductions similar to the cohorts who received 0.3 mg, but smaller reductions than the cohorts who received 1 mg or 2 mg. Therefore, the dose-escalation protocol was used in all subsequent clinical trials. No TEAEs of clinical concern or dose-limiting toxicities were observed [112].

Another phase I, randomized, double-blind trial examined the cardiac safety of ozanimod in healthy participants (*n* = 124) (Table 1). The dose of ozanimod was escalated to 2 mg, serving as a supratherapeutic dose in the treatment group. The primary endpoint was the Fridericia-corrected QT (QTcF) interval. The time-matched differences in change from baseline in QTcF for ozanimod compared to placebo (ΔΔQTcF) were 0.3 to 4.5 milliseconds for the therapeutic dose of ozanimod and −0.9 to 2.9 milliseconds for the supratherapeutic dose of ozanimod. One participant treated with ozanimod 2 mg and one treated with placebo had a QTcF > 450 ms; no participants had a QTcF > 480 ms. In ozanimod-treated patients, the mean hourly heart rate was consistently lower than in placebo-treated patients, with no further decrease in heart rate observed when increasing ozanimod from 1 mg to 2 mg. No clinically significant effects on the PR or QRS intervals were reported. The most common TEAE reported was a site reaction caused by ECG electrode tape. The incidence of cardiac TEAEs was comparable and infrequent in both study groups. These included short periods of nonsustained ventricular tachycardia (three placebo patients, two ozanimod patients), transient first-degree AV block (one ozanimod patient), and transient second-degree AV block Mobitz type 1 (one placebo patient, one ozanimod patient). This study determined that ozanimod did not prolong the QTc interval at therapeutic or supratherapeutic doses and did not demonstrate any new safety concerns [114].

### 11.2. Phase II Studies: RADIANCE Trial

The RADIANCE phase II trial was a 24-week randomized, double-blind, placebo-controlled clinical trial. Patients with relapsing MS (*n* = 258) were randomized to ozanimod 0.5 mg, ozanimod 1 mg, or placebo (Table 1). The primary endpoint was the cumulative number of gadolinium-enhancing lesions on MRI at weeks 12–24. The cumulative mean number was 1.5 (SD 3.7) in the ozanimod 0.5 mg group and 1.5 (SD 3.4) in the ozanimod 1 mg group, compared with 11.1 (SD 29.9) in the placebo-treated group. Patients in both ozanimod groups had fewer total gadolinium-enhancing lesions at week 24 and a smaller cumulative number of new or enlarging T2 lesions at weeks 12–24 when compared to placebo.

Sixty percent of participants (*n* = 156) reported at least one TEAE, with nasopharyngitis and headache being the most common. Orthostatic hypotension was the most frequent cardiovascular TEAE, with most events occurring on day 2 and all resolving spontaneously. There were no cases of macular edema, notable infectious, malignancy-related, or pulmonary adverse events. Second-degree AV block type 1 occurred in 2% (*n* = 4) of ozanimod-treated patients and 2% (*n* = 2) of placebo-treated patients; short sinus pause occurred in 1% (*n* = 1) of ozanimod-treated patients and 1% (*n* = 1) of placebo-treated patients. No cases of second-degree type 2 or third-degree AV block were reported. Three participants treated with ozanimod had an increase in ALT > 3 times the upper limit of normal (ULN), which resolved spontaneously. Circulating absolute lymphocyte count maximally decreased by 50% with ozanimod 0.5 mg group versus 59% with ozanimod 1 mg. The study concluded that both high and low dose Ozanimod was well tolerated with no evident difference between the doses [115].

After the RADIANCE phase II study concluded, participants could enroll in a dose-blinded extension study (Table 1). Participants previously treated with ozanimod continued treatment with the same dose, and participants previously assigned placebo were randomized to ozanimod HCl 0.5 or 1 mg. The 2-year extension study maintained the same efficacy endpoints as the RADIANCE phase II trial and was completed by 89.6% (223/249) of participants. The mean number of gadolinium-enhancing lesions remained low in ozanimod-treated patients in both study periods and decreased in the placebo to the ozanimod group. Before starting the extension, the proportions of participants free of gadolinium-enhancing lesions was 84.7% for the group continuing ozanimod HCl 0.5 mg, 87.7% for the group continuing ozanimod HCl 1 mg, 58.5% for the placebo to ozanimod HCl 0.5 mg group, and 69.0% for the placebo to ozanimod HCl 1 mg group. The proportions of participants free of gadolinium-enhancing lesions were 91.1–92.9% at the end of the first year and 86.5–94.6% at the end of the second year of the extension period. There was a dose-dependent reduction in the mean number of new or enlarging T2-hyperintense lesions on brain MRI during each year of the extension period. The unadjusted ARR was 0.32 for the group continuing ozanimod HCl 0.5 mg, 0.18 for the group continuing ozanimod HCl 1 mg, 0.30 for the placebo to ozanimod HCl 0.5 mg group, and 0.18 for the placebo to ozanimod HCl 1 mg group. The mean change in Expanded Disability Status Scale (EDSS) from baseline of the extension study was 0.2 for the group continuing ozanimod HCl 0.5 mg, 0.1 for the group continuing ozanimod HCl 1 mg, 0.3 for the placebo to ozanimod HCl 0.5 mg group, and 0.2 for the placebo to ozanimod HCl 1 mg group at the end of the second year.

TEAEs were reported by 78.6% (*n* = 99) of participants in the ozanimod HCl 0.5 mg group and 75.6% (*n* = 93) of participants in the ozanimod HCl 1 mg group. The most common documented TEAEs were nasopharyngitis, upper respiratory tract infection, and increased ALT. Four participants experienced an increase in ALT or AST levels >5 times the ULN, after which treatment was discontinued. No cases of serious opportunistic infection, clinically significant abnormalities in pulmonary function tests, macular edema, or malignancy were reported. There were no cases of clinically significant bradycardia or second-degree or higher AV block [99]. The maximum mean decrease in heart rate from the study baseline was 0.6 bpm, occurring on the first day of dose escalation in the placebo to ozanimod 0.5 mg group. No mean change in heart rate in the groups continuing treatment was reported.

### 11.3. Phase III Studies: RADIANCE Trial

The RADIANCE phase III trial was a 24-month double-blind, double-dummy, active-controlled, parallel-group clinical trial comparing daily oral ozanimod 0.5 mg or 1 mg with weekly intramuscular interferon beta-1a 30 μg, with 1320 participants with relapsing multiple sclerosis enrolled (Table 2). The primary endpoint was adjusted annualized relapse rate. The adjusted ARR was 0.17 for participants receiving ozanimod 1 mg, 0.22 for participants receiving ozanimod 0.5 mg, and 0.28 for participants receiving interferon beta-1a. The mean number of new or enlarging T2 brain lesions on MRI over the study period was 1.84 for the ozanimod 1 mg group, 2.09 for the ozanimod 0.5 mg group, and 3.18 for the interferon beta-1a group. The mean number of gadolinium-enhancing brain lesions on MRI was 0.18 for the ozanimod 1 mg group, 0.20 for the ozanimod 0.5 mg group, and 0.37 for the interferon beta-1a group at 24 months. Additionally, the study found that loss of whole-brain volume, cortical grey matter, and thalamic volume was reduced in both ozanimod doses when compared to interferon beta-1a. This study concluded that both doses of ozanimod were at least as effective as interferon beta-1a, and that the ozanimod 1 mg dose showed greater efficacy than the 0.5 mg dose.

TEAEs were reported by 74.7% (*n* = 324) of participants in the ozanimod 1 mg group, 74.3% (*n* = 326) of participants in the ozanimod 0.5 mg group, and 83.0% (*n* = 365) of participants in the interferon beta-1a group. The most common TEAEs in the ozanimod groups were nasopharyngitis, ALT increase, hypertension, γ-glutamyltransferase increase, pharyngitis, and urinary tract infection; the most common TEAEs in the interferon beta-1a group were influenza-like illness, headache, nasopharyngitis, upper respiratory tract infection, pyrexia, and orthostatic hypotension. TEAEs that led to discontinuation in the ozanimod groups included ALT increase, urticaria, and γ-glutamyltransferase increase; TEAEs that led to discontinuation in the interferon beta-1a group included influenza-like illness, ALT or AST increase, and macular degeneration. The incidence of TEAEs that led to discontinuation was higher in the interferon beta-1a group. There were no clinically significant cardiac TEAEs, no cases of second or third-degree AV block, and no serious opportunistic infections. Macular edema occurred in the interferon beta-1a group (*n* = 2) and the ozanimod 0.5 mg group (*n* = 2). Malignancies occurred in participants treated with ozanimod 1 mg (*n* = 4), ozanimod 0.5 mg (*n* = 3), and interferon beta-1a (*n* = 2), with no specific risk, identified. These trial findings demonstrated that ozanimod was well tolerated overall among participants with relapsing MS [98].

### 11.4. Phase III Studies: SUNBEAM Trial

The SUNBEAM phase III trial was a 12-month double-blind, double-dummy, active-controlled, parallel-group clinical trial, also comparing daily oral ozanimod 0.5 mg or 1 mg with weekly intramuscular interferon beta-1a 30 μg, with 1346 participants enrolled (Table 2). The adjusted annualized relapse rate was the primary endpoint. The adjusted ARR was 0.18 for the ozanimod 1 mg group, 0.24 for the ozanimod 0.5 mg group, and 0.35 for the interferon beta-1a group.

The mean number of new or enlarging T2 brain lesions on MRI over the study period was 1.47 with ozanimod 1 mg, 2.14 with ozanimod 0.5 mg, and 2.84 with interferon beta-1a. The mean number of gadolinium-enhancing brain lesions on MRI was 0.16 with ozanimod 1 mg, 0.29 with ozanimod 0.5 mg, and 0.43 with interferon beta-1a at month 12. As in the RADIANCE phase III trial, this study reported a reduction in the loss of whole-brain volume, cortical grey matter, and thalamic volume with ozanimod when compared to interferon beta-1a. SUNBEAM also concluded that both low and high-dose ozanimod was as effective as interferon beta-1a in reducing active disease in relapsing MS, with ozanimod 1 mg demonstrating numerically greater efficacy than ozanimod 0.5 mg [97]. A pooled analysis of RADIANCE phase III and SUNBEAM found that the proportion of participants with disability progression at 3 and 6 months did not significantly differ between treatment groups [98].

TEAEs were reported by 59.8% (*n* = 268) of participants in the ozanimod 1 mg group, 57.2% (*n* = 259) participants in the ozanimod 0.5 mg group, and 75.5% (*n* = 336) of participants in the interferon beta-1a group. The most common TEAEs in participants treated with ozanimod were nasopharyngitis, headache, and upper respiratory tract infection. The most common TEAEs in participants treated with interferon beta-1a were influenza-like illness, nasopharyngitis, pyrexia, headache, and upper respiratory tract infection. The most common TEAEs leading to discontinuation included influenza-like illness, back pain, headache, and ALT increases. TEAEs leading to treatment discontinuation occurred in 13 participants from the ozanimod 1 mg group, 7 from the ozanimod 0.5 mg group, and 16 from the interferon beta-1a group. There were no cases of serious opportunistic infections or second or third-degree AV block reported. One serious TEAE of asymptomatic sinus bradycardia occurred that resolved spontaneously.

An ALT >3 times the ULN was documented in 4.3% (*n* = 19) of patients in the ozanimod 1 mg group, 1.8% (*n* = 8) in the ozanimod 0.5 mg group, and 2.2% (*n* = 10) in the interferon beta-1a group; most cases were transient and resolved spontaneously. Macular edema occurred in one interferon beta-1a treated patient, and two ozanimod-treated patients (both with predisposing factors). Malignancies occurred in both the ozanimod 1 mg (0.2%, *n* = 1) and ozanimod 0.5 mg (0.4%, *n* = 2) groups; there were no malignancies reported in participants treated with interferon beta-1a. This study demonstrated that ozanimod is well tolerated, with a lower incidence of TEAEs when compared with interferon beta-1a. A long-term, open-label extension study for RADIANCE and SUNBEAM study participants is currently active (NCT02576717) [97].

### 11.5. Additional Studies

A comparative review used data from the RADIANCE phase III and SUNBEAM clinical trials to compare the safety and efficacy of ozanimod versus fingolimod 0.5 mg, using data from the phase III trials TRANSFORMS, FREEDOMS, and FREEDOMS II (Table 2). Adjusted analyses of both 1-year and 2-year outcomes found no significant difference in ARR between groups. The 1-year ARR ratio was 1.08 (*p* = 0.80), and the 2-year ARR ratio was 1.06 (*p* = 0.78). At two years, the proportions of participants free of 3-month and 6-month confirmed disability progression was similar between ozanimod and fingolimod. In a comparison of first-dose cardiac monitoring outcomes, the adjusted risk difference (RD) was favorable for ozanimod versus fingolimod. The RD was −3.5% for conduction abnormalities, −3.0% for first-degree AV block, and −8.3% for monitoring necessary beyond 6 h for ozanimod compared with fingolimod (all *p* < 0.001). Ozanimod showed a significantly lower risk of any adverse event compared to fingolimod (RD −9.9%), and had a lower risk of TEAE leading to treatment discontinuation in the 2-year safety outcomes [116].

A presentation at the American Academy of Neurology reviewed data on pregnancy outcomes in ozanimod-treated participants in clinical trials. Exposure to ozanimod was limited to the first trimester as participants were required to discontinue the study treatment. A total of 21 pregnancies were reported; resulting in 13 live births, 2 spontaneous abortions, and 7 elective terminations. All live births resulted in healthy full-term newborns, with no evidence of teratogenicity observed and no signal of adverse pregnancy outcomes [117].

## 12. Conclusions

Our understanding of multiple sclerosis pathophysiology and its clinical implications has grown tremendously over the decades. Treating the disease has become a complex task as clinicians now have many pharmacologic treatment options for this indication. Current therapeutic options include three broad categories: management of acute relapse, disease modifying therapies (DMTs), and symptomatic management. Clinicians should also be aware of the off-label use of medications to treat MS, such as Rituximab and neuromodulation techniques. Many of the aforementioned therapies were not originally developed to target MS pathophysiology and as such prove insufficient alone, highlighting the need for targeted MS treatments with reduced systemic side effect profiles.

Ozanimod is recently FDA approved for the treatment of clinically isolated syndrome, relapsing–remitting, and secondary progressive forms of MS. It is an oral agent that selectively modulates S1P1 and S1P5 receptor activity, which prevents autoreactive lymphocytes from entering the CNS where they can promote nerve damage and inflammation. This selectivity allows for modification of the disease course and once-daily dosing, and contributes to lower systemic side effects when compared to other drugs of its class. Furthermore, Ozanimod is currently the only US FDA approved S1PR modulator that does not require first-dose observation or genetic testing. Numerous clinical studies have demonstrated that ozanimod is both effective in the treatment of multiple sclerosis and well tolerated by patients. Further studies are required to evaluate its efficacy when compared with other available therapies, and as our understanding of MS pathophysiology and disease progression continues to evolve.

## Figures and Tables

**Table 1:** Clinical Efficacy and Safety.

Author (Year)	Groups Studied and Intervention	Results and Findings	Conclusions
Tran J. et al. (2017) [112]	Phase 1 single-center, randomized, double-blind, placebo-controlled study comparing single-ascending doses of ozanimod 0.3, 1, 2, or 3 mg; 7-day multiple ascending-doses of ozanimod 0.3, 1, or 2 mg; 28-day multiple ascending-doses of ozanimod of 0.3, 1, or 1.5 mg; a dose-escalation protocol up to ozanimod 2 mg; and placebo in 88 healthy subjects.	A dose-dependent negative chronotropic effect occurred on day 1 with ozanimod. This effect was mitigated in the dose-escalation cohort.	The dose escalation protocol appears to be a safer approach to dosing and has been carried forward into subsequent clinical trials.
Tran J. et al. (2018) [114]	Phase 1 single-center, randomized, double-blind, placebo-controlled, positive-controlled, parallel-group thorough QT/QTc study comparing ozanimod 0.25, 0.5, 1, and 2 mg to placebo in healthy subjects.	One ozanimod-treated subject and one placebo-treated subject had a QTcF > 450 ms; no subjects had a QTcF > 480 ms. There were no clinically significant effects on the PR or QRS intervals. The incidence of adverse effects was similar between ozanimod-treated and placebo-treated groups.	Ozanimod does not prolong the QTc interval at therapeutic or supratherapeutic doses. There were no safety issues discovered during this study.
Cohen J. et al. (2016) [115]	Phase 2 multi-center, randomized, double-blind, placebo-controlled clinical trial (RADIANCE) comparing ozanimod 0.5 and 1 mg with placebo in subjects with relapsing multiple sclerosis over 24 weeks.	The mean cumulative number of gadolinium-enhancing lesions on MRI was reduced with both doses of ozanimod: 1.5 with ozanimod 0.5 mg and 1.5 with ozanimod 1 mg versus 11.1 with placebo. The most common TEAEs were nasopharyngitis and headache. There were no serious infectious or cardiac adverse events and no cases of macular edema.	Ozanimod was effective in reducing MRI lesion activity and was well tolerated in participants with RRMS.
Cohen J. et al. (2019) [99]	Dose-blinded 2-year extension of the RADIANCE phase 2 study; participants previously assigned ozanimod continued at the same dose and participants previously assigned placebo were randomized to ozanimod 0.5 or 1 mg.	The number of gadolinium-enhancing lesions and new or enlarging T2 lesions were low in all treatment groups throughout the study period. The TEAEs reported in this study were consistent with those seen during the 24-week RADIANCE phase 2 study. There were no clinically significant cardiac TEAEs. There were four cases of increased ALT that led to study discontinuation; all recovered after drug cessation.	Ozanimod demonstrated continued efficacy in participants previously assigned ozanimod and reached similar efficacy in participants who were previously assigned placebo. Ozanimod continued to be well tolerated with no safety issues discovered. The incidence of TEAEs did not appear to increase over time and was similar between the two doses.

**Table 2:** Comparative Studies.

Author (Year)	Groups Studied and Intervention	Results and Findings	Conclusions
Cohen J. et al. (2019) [98]	Phase 3 multi-center, double-blind, double-dummy, active-controlled, parallel-group clinical trial (RADIANCE) comparing ozanimod 0.5 and 1 mg with interferon beta-1a 30 μg in subjects with relapsing multiple sclerosis over 24 months.	The adjusted annualized relapse rate at 24 months was 0.17 with ozanimod 1 mg, 0.22 with ozanimod 0.5 mg, and 0.28 with interferon beta-1a. Ozanimod was also associated with significantly lower numbers of gadolinium-enhancing lesions and new or enlarging T2 lesions than interferon beta-1a. The incidence of TEAEs was higher in the interferon beta-1a group than in either ozanimod group. There were no clinically significant cardiac TEAEs, and the incidence of infection was similar across treatment groups.	Both doses of ozanimod were more effective than interferon beta-1a in clinically meaningful measures of disease activity. The ozanimod 1 mg dose showed numerically greater efficacy than the 0.5 mg dose. Ozanimod was well tolerated in this study.
Comi G. et al. (2019) [97]	Phase 3 multi-center, double-blind, double-dummy, active-controlled, parallel-group clinical trial (SUNBEAM) comparing ozanimod 0.5 and 1 mg with interferon beta-1a 30 μg in subjects with relapsing multiple sclerosis over 12 months.	The adjusted annualized relapse rate at 12 months was 0.18 with ozanimod 1 mg, 0.24 with ozanimod 0.5 mg, and 0.35 with interferon beta-1a. There were significantly lower numbers of gadolinium-enhancing lesions and new or enlarging T2 lesions with ozanimod than with interferon beta-1a. The incidence of TEAEs was higher in the interferon beta-1a group than in either ozanimod group. There were no clinically significant cardiac TEAEs, and the incidence of infection was similar across treatment groups.	Both doses of ozanimod were more effective than interferon beta-1a in reducing active disease. The ozanimod 1 mg dose showed numerically greater efficacy than the 0.5 mg dose. Ozanimod was well tolerated in this study.
Swallow E. et al. (2020) [116]	Comparative review using published data from the phase 3 clinical trials RADIANCE, SUNBEAM, TRANSFORMS, FREEDOMS, and FREEDOMS II to compare the safety and efficacy of ozanimod with fingolimod 0.5 mg.	There was no significant difference in annualized relapse rate or proportions of participants free of confirmed disability progression at 3 and 6 months between ozanimod and fingolimod. Ozanimod showed favorable safety outcomes when compared to fingolimod, including a significantly lower risk of any adverse event.	Efficacy outcomes were similar between ozanimod and fingolimod. Ozanimod was associated with a more favorable benefit-risk profile than fingolimod.
